# Qualitative evidence for improved caring, feeding and food production practices after nutrition-sensitive agriculture interventions in rural Vietnam

**DOI:** 10.1186/s40066-021-00350-5

**Published:** 2022-04-11

**Authors:** Dai Dinh Nguyen, Sabina Di Prima, Reint Huijzendveld, E. Pamela Wright, Dirk Essink, Jacqueline E. W. Broerse

**Affiliations:** 1 Athena Institute, Vrije Universiteit Amsterdam, 1081HV Amsterdam, The Netherlands; 2 Faculty of Science, Vrije Universiteit Amsterdam, Amsterdam, The Netherlands; 3 Guelph International Health Consulting, Amsterdam, The Netherlands; 4 Medical Committee Netherlands-Vietnam (MCNV), Block 3, Dong Luong ward, Dong Ha, Quang Tri province Vietnam

**Keywords:** Nutrition-sensitive agriculture, Behaviour change, Impact pathways, Ethnic minority groups, Remote areas, Process assessment

## Abstract

**Background:**

Research on nutrition-sensitive agriculture (NSA) has mostly been aimed at demonstrating its impact on nutrition and explicating underlying pathways, and more rarely at understanding processes and lessons learnt from them. This study aimed to gain insights into the processes that influence behaviour change, contributing to improved caring, feeding and food production practices, using a program theory perspective. It also investigated perceived challenges to the sustainability of interventions and potential solutions, in the context of an NSA program in rural Vietnam. Using a participatory approach, data were gathered on impact pathways and perceived outcomes, on elements of program theory that led to behavioural change, as well as barriers and facilitators. Respondents in semi-structured interviews (*n* = 30) and seven focus group discussions (total *n* = 76) were selected purposively among program participants. Data was collected and triangulated across several stakeholder groups.

**Results:**

The impact pathways (production-consumption, caring and feeding, and home-grown school feeding) envisaged in the NSA program functioned as intended; synergies were revealed. The increased supply of locally produced nutrient-rich foods not only contributed to the emergence of a promising income sub-pathway but also reinforced synergy with the home-grown school feeding pathway. Improved diets, feeding and caring practices, and school attendance were key outcomes of the program. Successful elements were pathway-specific, such as flexibility in implementing context-appropriate agricultural models. Others, such as benefit-driven motivation and improved knowledge, triggered changes in multiple pathways. Role models, increased self-confidence, and change agents were the main process facilitators. The biggest barrier to both implementation and sustainability was the poor socio-economic conditions of the most disadvantaged households.

**Conclusions:**

This study showed the relevance of NSA programs in addressing undernutrition in remote areas by enhancing self-reliance in local communities. The integration of behaviour change activities proved to be a key strategy in the process to enhance the impact of agriculture on nutrition outcomes. Though outcomes and influencing factors are very context-dependent, lessons on what worked and what did not work could inform the design and implementation of effective behaviour change strategies in future NSA programs in Vietnam and elsewhere.

## Background

Despite significant improvements in food security and nutrition over the last decades, undernutrition remains a major health and development challenge, raising doubts about the prospect of achieving the Sustainable Development “zero hunger” goal by 2030 [1, 2]. Globally, about 149 million children under 5 years of age (CU5) are stunted and 49 million are wasted [3]. Almost half of under-five child deaths (about 3.1 million children per year) are caused by undernutrition [4]. Micronutrient deficiencies are also quite prevalent in this age group; for example, about 29% of CU5 in low- and middle-income countries are vitamin A deficient [5]. Child undernutrition results from the interplay of multiple causes, including poverty, insufficient dietary intake and diversity, inadequate care and feeding, lack of a hygienic living environment, and limited access to health services [4]. In the first 1000 days from conception, undernutrition increases child morbidity and mortality [4], with a high risk of dying even from common infections [6]. Furthermore, undernutrition in childhood affects physical and cognitive development, and ultimately earning potential and reproductive outcomes as adults, perpetuating an intergenerational vicious cycle of inadequate nutrition, poor health and low productivity [7–9].

Even in Asian countries with substantial economic progress, such as Vietnam, undernutrition persists as a problem [10, 11]. The burden is particularly significant among disadvantaged ethnic minority groups in remote mountainous areas, where socio-cultural, agro-ecological and climate-related factors exacerbate the problem [11, 12]. In Vietnam, the highest stunting rates occur among CU5 in ethnic minority communities, and similar patterns are observed for underweight and micronutrient deficiencies [11]. Ethnic minorities represent a significant segment of the Vietnamese population (14%, approximately 13.5 million people),  so that undernutrition among them has considerable social and economic impact [11]. The relevance of the problem is even more striking when accounting for the global mountain population estimated to be 915 million people, of which 90% live in developing countries [13]. Among them, nearly 329 million were considered vulnerable to food insecurity in 2012 [13].

Much emphasis has been placed on nutrition-specific interventions, such as micronutrient supplementation, that aim to address the direct determinants of children’s nutritional status. However, there is growing consensus that such interventions cannot solve persistent undernutrition on their own [14]. The multi-faceted nature of the problem calls for multi-sectoral approaches involving complementary implementation of nutrition-sensitive programs [15, 16]. These are expected to improve the underlying determinants of nutrition security (food security, care practices, health services and hygienic environment) and, to a certain extent, basic determinants (poverty and access to resources) at household and community level [17]. Nutrition-Sensitive Agriculture (NSA) programs, in particular, have strong potential to improve nutrition security because of their multiple pathways simultaneously influencing nutrition outcomes [15, 18, 19]. They hold promise especially in rural areas, where agriculture is central to the provision of food, livelihoods and income [15]. NSA could also play a role in addressing key priorities in sustainable agriculture production, but will need to be combined with strategies to decrease food loss and waste [20–22].

In line with past NSA-related reviews [23, 24], a recent study confirmed that the evidence of impact of NSA interventions on nutritional status based on anthropometric indicators is limited [25]. The same study validated how such interventions can improve food access, dietary and care practices and, to some extent, children’s micronutrient status [25]. However, the outcomes of NSA interventions are influenced by a wide range of factors at different levels [26].

NSA *“aims to improve nutrition by strengthening agricultural systems to deliver more appropriate, nutritious foods to those needing them”* [27]. NSA programs incorporate specific nutrition objectives and operationalise them through integrated intervention packages often including agriculture, nutrition education, gender sensitisation/women’s empowerment and water, sanitation and hygiene (WASH) as core components [24]. The integration of a strong behaviour change component to promote improved diets and child care and feeding practices is consistently reported as a key strategy to enhance the impact of agriculture on nutrition outcomes through the knowledge pathway [24, 25]. Although positive effects on knowledge and practices were observed in past programs, behaviour change strategies could be further improved to maximise NSA potential [24]. There is a need to identify best practices in the design and implementation of effective and affordable behaviour change strategies, learning how to increase their value for beneficiaries without excessive increases in workload [24]. Furthermore, since the level of complexity increases in multi-sectoral NSA programs, selecting which behaviours to prioritise and which mechanisms to apply is a challenge, as nutrition outcomes are the cumulative effect of interlinked changes on multiple dimensions (production, food purchasing, consumption, caring and feeding, among others) [28]. As Green and Kreuter stated, *“people learn continuously from their environmental and social surroundings and can develop, individually or collectively, the knowledge and skills to modify them”* [29].

To address gaps in our knowledge about how these changes evolve in an NSA program, and to propose potential strategies for future programs, this study aimed to gain insights into the processes that influence why and how behaviour change triggers and contributes to improved caring, feeding and food production practices, using a program theory perspective. It also investigated perceived challenges to the sustainability of interventions and potential solutions, in the context of an NSA program in rural Vietnam.

### Program description

To address the undernutrition challenge among ethnic minority groups, the Medical Committee Netherlands-Vietnam (MCNV), with Phu Yen province and Dong Xuan district authorities, started a four-year NSA program in a mountainous commune in 2017. The program aimed at reducing undernutrition by improving food access and food intake for CU5. It was a combined implementation-research NSA program, in cooperation with Hue University of Agriculture and Forestry, Hue University of Medicine and Pharmacy, and the Vrije Universiteit Amsterdam. The situation in Phu Mo commune before the start of the project is summarised in the box below.

Box 1 Phu Mo commune, situation at baseline These data were collected during the baseline study in March 2018 in Phu Mo commune, covering 224 households unless otherwise indicated.

- Most of the Cham and Bana ethnic minority people (63%) lived below the Vietnamese poverty line (from government data);
- Food insecurity was common: using the Food Insecurity Experience Scale, among 224 households, only 13% were food secure. Among the majority, 42% were mildly food insecure, 39% moderately and 6% severely food insecure;
- Children under five (*n* = 243) were often underweight (43%) and/or stunted (61%);
- Most (87%) households were dependent on income from farm work, planting and harvesting cash crops, such as cassava and acacia;
- Almost half of households (46%) focused on cash-crops, especially cassava, with an unreliable price that has steadily declined since 2012;
- Rice was another key crop (wet paddy: 33%; upland: 31%), but nearly all rice (96%) was consumed at home;
- Only half of the households raised chicken, using the meat but not the eggs, and only an average of four birds per household;
- Fewer than half of the households had home gardens (40%), often used to grow tobacco. Mustard greens were cultivated by only about 6% of the households, during two months/year. Gourds and bananas were seldom seen. Most families did grow eggplants in cassava or upland rice fields;
- The commune was highly dependent on external food supplies. They grew their own rice but bought other foods mainly from mobile vendors. More than half (52%) continued to collect wild foods in the nearby forests, both wild vegetables, and rats;
- People purchased the following items from mobile vendors or nearby markets: fish, meat and eggs (87% of households), vegetables (79%), sugar and salt (78%), rice when their own production was insufficient (63%), and milk (30%);
- Products sold in local shops were often dried and packaged, such as instant noodles, porridge and snacks;
- Most families consumed rice, cassava leaves, wild vegetables, chili and salt on a daily basis;
- Children’s dietary diversity was low: 24-h recall revealed that among 243 CU5, 24% had consumed only one food group and 60% only two, both predominantly grains. Very few (less than 5%) reported consuming all of the following food groups in a day: 'starchy staples', 'meat and fish', 'fruits and vegetables' and ‘dairy products’;
- Nursery schools in the ethnic minority villages did not provide meals; none of those in Phu Mo and only one of the 16 in Dong Xuan district did that before the school year 2017–2018;
- Daily attendance at nursery schools when the project started was high at 88% (school attendance is compulsory);
- When the project started, no other projects were being implemented or even just completed, neither by Government or by NGOs; this NSA program was the first intervention in this commune.

The NSA integrated package involved three key sectors: agriculture, health and education. All sectors contributed to program design and implementation, but each took responsibility to spearhead and monitor specific components. The progressive assessment of results and improvements of design based on lessons learnt were a cross-sectoral effort. Three main entry points addressed undernutrition: context-appropriate agricultural models (key entry point), health and nutrition education, and home-grown school feeding (HGSF). Unlike many NSA programs, the package did not include women’s empowerment; the targeted ethnic minority groups follow matriarchal traditions and women have significant decision-making power.

The main objectives of the **agricultural component **were to improve homestead food production and promote a partial shift from cash crops to nutritious crops, targeting 150 of the 300 households with CU5 and/or pregnant women in Phu Mo commune. The beneficiaries of this component were selected by the twenty household groups in which the participating 300 households were organised. Households with malnourished CU5 as well as poor and most disadvantaged households were considered eligible. Beneficiary households and commune agricultural staff received in-field trainings on nutrition-sensitive production systems integrating vegetables, fruit and poultry, plus sustainable land management techniques, such as intercropping and agroforestry. Households were given a one-time small grant of USD 172 to 229 to establish the improved systems; these households have an income up to USD 175 per month. Exchange visits among beneficiary farmers of the five villages were organised to facilitate sharing of experiences.

The **health-nutrition component** aimed at improving caring and feeding practices and access to health services. All 300 households with CU5 and/or pregnant women were targeted, 150 of which also participated in the agricultural interventions. Staff of commune health stations and village health workers (VHWs) were trained on early detection and treatment of severely malnourished children and pregnant women and on nutrition counselling. Local health staff was also trained on disease prevention, growth monitoring, hygiene, and breastfeeding. The leaders of the twenty household groups mentioned above received hands-on training at the community centres. They acquired knowledge and skills to facilitate the monthly household group meetings (HGMs) held in their own homes, where experience was shared about caring, feeding and health practices, cooking demonstrations, growth monitoring, and peer-to-peer support. They received a compensation of about 50% of their daily labor wage (approximately USD 3.5/day). The group leaders were given communication materials and measuring scales to facilitate discussions.

The primary objectives of the **HGSF component** were to provide nutritious school meals to all five nursery schools by 2020 and to establish sustainable local micro-enterprises to supply the meals. The activities targeted all children registered at the nursery schools. Four female micro-entrepreneurs, with good cooking skills and business propensity, were selected and trained on food preparation, hygiene and safety, and financial management by staff of the Department of Education and Training. The latter provided them with monthly menus designed according to the national school menu framework of the Ministry of Education. Micro-entrepreneurs procured the ingredients from both external and home-grown sources: the district market, mobile vendors, own production and local farmers. To give them a boost and an incentive, micro-entrepreneurs received basic equipment: refrigerator, blender, rice cooker and food containers. Financial support was provided only during the two-week training period before supplying school meals. To ensure safety of school feeding, nursery school teachers were trained on nutrition, feeding, and WASH practices and given responsibility for monitoring the supply of daily menus. Teachers provided nutrition and WASH education to mothers who volunteered to help with daily feeding at breakfast and lunch. To stimulate community buy-in to the HGSF component, the NSA program fully subsidised the first month’s pilot (about USD 0.65/child/day). After that, parents contributed 33% of the costs. The government’s monthly subsidy to parents for school meals covered about 45% of the cost. From January 2020, the parents’ contribution increased to 50%; from 2021 they were expected to fully cover the costs.

## Methods

### Study area

The study was conducted at the NSA program site in Phu Mo commune, Dong Xuan district, Central Vietnam. Phu Mo commune, which literally translated means “rich and fertile”, is the most remote and poor commune in the southern central province of Phu Yen. It comprises five villages (Phu Tien, Phu Hai, Phu Giang, Phu Dong and Phu Loi), where over 3200 people of the Cham and Bana ethnic minorities live. Box 1 describes the situation at the start of the project. Unstable market prices for cassava, their main cash crop, inadequate supply of nutritious food, and limited knowledge of nutrition, childcare and feeding practices are among the determinants of their persistent food security and nutrition problems. Additional socio-demographic information about the population in the study area is presented in Box 2.

Box 2 Socio-demographic information about Phu Mo population

- The commune counts 837 households (about 3,250 persons) distributed in five villages with a population density of approximately 7 people per km^2^;
- 99% of the population belongs to the Bana and Cham ethnic minority groups;
- 44% of the households are poor with a monthly income below USD 120/household; within this group, there are the very poor (about 5%) who often have very little or no agricultural land. The relatively poor households account for 40% of Phu Mo population. They have a monthly income below USD 175/household;
- Most of the inhabitants (70%) have completed secondary school (grade 6 to 9, 12 to 15 years old). The percentage of illiterate people or with only primary schooling is small (5.5%);
- The average land size in these upland villages is 0.38 hectares per household, which is less than the government standard of 0.5

### Study design

**Fig. 1:**
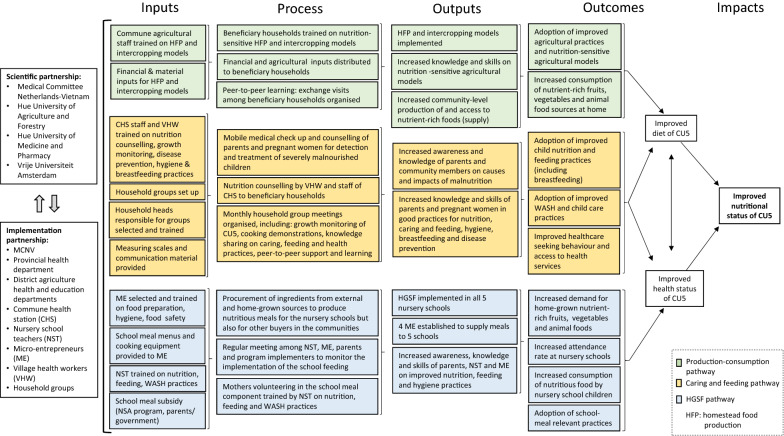
Ex-ante program theory of change

A participatory research approach was used to gather qualitative data on impact pathways and perceived outcomes, and elements of the program theory that led to behavioural change in relation to caring, feeding and agricultural practices, as well as on barriers and facilitators. Opportunities and challenges for program sustainability were identified. We used the following definitions according to the program theory (Fig. 1):

- ***Outcomes:*** the main changes (intended or not) that the participants observed in the study area and the targeted population since the implementation of the NSA interventions. Outcomes can be influenced through intervention design factors/strategies, but as stated by Lodenstein et al. [30] *“The outcomes of interventions are highly dependent on human agency and context, and interventions do not linearly produce outcomes”*.
- ***Successful elements of program theory:*** input, process and output elements leading to success in the perception of the respondents. They capture the why and how change occurred at individual, household and/or community level as a result of program decisions.
- ***Facilitators and barriers:*** those factors that enabled and/or constrained the implementation of the NSA program. Such factors are usually related to the cultural, socio-economic, bio-physical context in which the interventions are implemented. Contextual factors are less easily influenced and, in the context of an intervention, are often considered preconditions. However, implementers have more influence on these factors if they are internalized as part of program design (intervention design factors), for example, the role models and change agents in our case.

The overall methodological approach for this study was largely based on past experiences with theory-driven process evaluation [31]. A first step in the study design was to construct an ex-ante program theory of change based on secondary data (program internal reports) to map the envisaged primary pathways of change: production-consumption, caring and feeding, and HGSF. A detailed description of the program theory, including impact pathways and program processes, is presented in Fig. 1. This visualisation is a simplification of the complex reality in which most elements are interlinked, creating an intricate network of relations. The program theory was informed by existing conceptual frameworks on agriculture to nutrition pathways [18, 19, 32], which guided the design of data collection tools and data analysis. Starting from the program theory, an initial set of topics related to the themes of agricultural production and young child caring and feeding practices was developed, which formed the basis of tailor-made tools for each stakeholder group. The selection of topics considered the principles of the Precede–Proceed model, which describes behavioural change as a concurrence of predisposing, reinforcing and enabling factors, and assumes that environmental factors, such as social and physical factors often outside individual control, can be modified to support the envisaged change in behaviour [29]. The program theory was later used to unpack the primary impact pathways and explore emerging synergies across pathways.

### Study population

Data was collected and triangulated across several stakeholder groups at village, commune and district levels to enhance the validity of the qualitative findings. All five villages of Phu Mo commune were included. Study participants for the semi-structured interviews (SSIs) and focus group discussions (FGDs) were selected by purposive sampling. The overview of respondents and tools is presented in Table 1.

**Table 1:** Overview of respondents and data collection tools

Level	Respondents	SSIs	FGDs	FGD participants
Implementing NGO	Chief of office/lead implementer	1	–	–
District	Lead implementers from agriculture, health and education departments	3	1	7
Commune	Principal of Phu Mo nursery schools, health station representative, agriculture extension staff	3	1	5
Village	Village health workers (VHWs)	5	–	–
	Nursery school teachers	5	–	–
	Micro-entrepreneurs	4	–	–
	Parents/farmers (across the five villages)	9	5	64
Total		**30**	**7**	**76**

Implementers shared two main eligibility criteria: being directly involved in the NSA program from the start (2017) and being familiar with all components of the program. Respondents in the beneficiary group were selected and invited by the village leaders among members of households with CU5 and/or pregnant women actively participating in the program. Only beneficiaries involved in the full package of interventions (agriculture, health/nutrition and education) were invited for the FGDs. While the FGDs had been designed for parents, in practice only mothers attended, with a turnout between 9 and 18 per village. Background information on village level respondents is presented in Table 2.

**Table 2:** Socio-demographic information on village level respondents

Respondent groups	Socio-demographic information
Village health workers (VHWs)	● VHWs are local persons selected by the villagers and trained by the health sector ● All five VHWs of Phu Mo commune (3 males; 2 females) participated in the study ● Respondents’ average age was 32 years (range: 27 to 47) ● They had high school education (grade 10 to 12, 16 to 18 years old) and a nine-month training as VHW ● VHWs have no salary. They receive a monthly working allowance from the government (USD 6.6); VHWs also have a livelihood as farmers
Nursery school teachers	● Teachers are fulltime staff contracted by the district education department ● All five teachers of the Commune (5 female) participated in the study ● Respondents’ average age was 40 years (range: 35 to 52) ● They had high school education (grade 10 to 12, 16 to 18 years old) and a three-year training to become preschool teachers ● Average income was USD 264/person/month
Micro-entrepreneurs	● Micro-entrepreneurs are female villagers with small local businesses ● All four micro-entrepreneurs in the project participated in the study ● Respondents’ average age was 46 years (range: 35 to 56) ● They had high school education (grade 10 to 12, 16 to 18 years old) ● Average income was USD 287/person/month
Parents	● Data below refers to the 64 parents who participated in the five FGDs ● 100% of the participants were female ● Respondents’ average age was 28 years (range: 20 to 36) ● 73% had secondary school education (grade 6 to 9, 12 to 15 years old), 22% high school (grade 10 to 12, 16 to 18 years old) and 5% primary school (grade 1 to 5, 6 to 11 years old) or lower ● 50% of the respondents were poor (below USD 31/person/month), 28% were relatively poor (below USD 44/person/month) and 22% had an income above USD 44/person/month

### Data collection

The qualitative tools were developed in English by the research team then translated into Vietnamese. Data were collected between April and July 2020, 32 months after the start of the program. Given logistic constraints related to the COVID-19 quarantine, some interviews were done at a distance and video-recorded. All FGDs and most SSIs at village level were conducted face-to-face and audio-recorded. They were first transcribed into Vietnamese then translated into English; two researchers checked fidelity against the audio/video records. SSIs and FGDs were conducted by an experienced facilitator, together with a District Health staff member.

SSIs focused on program activities, perceived outcomes, processes through which outcomes were achieved, barriers and facilitators, sustainability of the program, and solutions to implementation challenges. SSIs followed guides tailor-made for each stakeholder group. After a pilot test, the interview guide was fine-tuned and shortened. SSIs were conducted iteratively to validate and build on the content of previous interviews as data collection progressed from district to village level. The investigators continued with the SSIs until saturation of data was reached and no new information emerged. In total thirty interviews took place, each lasting approximately 60 min.

FGDs were used to validate the findings from interviews and to gain deeper insights, particularly on pathways and their synergies and on elements contributing to success and sustainability. The same data collection tool was used for the FGDs at commune and district levels, while a simplified version was used in villages. The FGD tools comprised open-ended questions as well as visual prompts to facilitate the cross-sectoral discussion around the three primary impact pathways: production–consumption, caring and feeding, and HGSF. Five FGDs were held with in total 64 parents/farmers at village level and lasted about 60 min. The two FGDs at commune level (*n* = 5) and district level (*n* = 7) lasted longer (1.5–2 h).

To enhance the validity of qualitative findings, notes were taken and, when possible, verified with the respondents at the end of each FGD session. Summaries of individual interviews were used for member checking, while FGD findings were validated during commune and district meetings.

The results of the qualitative process assessment were triangulated with findings of the field observations and transect walks conducted regularly (monthly, except during the COVID quarantine period) by MCNV staff and District staff from agriculture, health and education.

### Data analysis

The English transcriptions of the SSIs and FGDs were used for thematic analysis. Transcripts were first summarized, then coded and analysed using the software program Atlas.ti 8. The codebook was developed through an iterative process involving all co-authors. While the overarching themes of the codebook were developed deductively, the list of codes was generated inductively. Codes emerged progressively during analysis and were used to categorize text segments from the SSIs and FGDs under the pre-defined themes. First, SSIs and FGDs were coded systematically and independently by one researcher. The others reviewed the raw data at various points during analysis and code attribution was subsequently discussed and agreed upon. The findings reflect the perspectives of the different stakeholder groups; specific quotations are used to illustrate key points. As respondents were anonymized, each quotation received a distinctive label comprising the respondent unique identifier and group category, for example “R1, District health”. The process was guided by the principles and strategies for qualitative data analysis of Bazeley [33]. The horizontal content analysis built on the program theory of change and was structured around the impact pathways. It provided insights on implementation and outcomes of interventions, the changes they stimulated, and how and why those changes occurred. The most important barriers and facilitators encountered, challenges for sustainability and potential solutions, and important synergies across the primary pathways were also covered.

### Ethical approval and consent to participate

The Institutional Ethics Committee of Hue University of Medicine and Pharmacy in Vietnam approved this study (registration number H2018/010.) Prior to the SSIs and FGDs, participants signed an informed consent form and authorized audio/video-recording.

## Results

The three primary impact pathways identified in the ex-ante program theory of change, production–consumption, caring and feeding, and HGSF, were validated and further unpacked through the SSIs and FGDs. In this section, we first present the findings for each pathway, with focus on the outcomes, elements of the program theory that led to success, facilitators and barriers. Then, we will report aspects related to the synergy across pathways. Finally, we will describe the sustainability challenges and potential solutions put forward by the respondents. The perception of stakeholders about the changes they noticed are summarised in Table 3. Although the findings are broadly similar, each group of respondents described what they experienced or observed from their own unique point of view.

**Table 3:** Perceptions about change among different stakeholders

Stakeholder group	District authorities	Commune authorities	Village health workers	Nursery school teachers	Micro-entrepreneurs	Parents/ farmers
Production–consumption pathway	● Multi-stakeholder interventions led to farmers’ behaviour changes; ● Role models and influential community members enhanced confidence; ● Partial shift from cash crops to non-cash nutritious crops; ● Increased diversity of nutritious crop production; ● At least 15% of farmers have surplus nutritious food to sell to communities and micro-entrepreneurs; ● Vulnerable households still focus on labour and cash crops for income.	● Partial shift from cash crops to non-cash nutritious crops; ● Increased diversity of nutritious crop production; ● Nutritious food integrated in household diets; ● Shift from buying food with wages to producing own food and selling surplus; ● Vulnerable households still focus on labour and cash crops for income.	● Increased food diversity (production and diet) and NSA practices; ● More nutritious daily food intake for children and pregnant women; ● Farmers will continue growing nutritious crops after project ends.		● Farmers produce more nutritious food for daily intake; ● Farmers sell surplus food to micro-entrepreneurs and communities.	● Increased food diversity (production and diet) and NSA practices; ● Farmers will continue growing nutritious crops, seeing good results; ● More confident and knowledgeable in implementing new NSA models; ● Vulnerable households still focus on labour and cash crops for income.
Caring–feeding pathway	● Intersectoral interventions supported at least half of families to change behaviour in caring and feeding for children and pregnant women: seeking health care, hygiene, breastfeeding, and vaccination; ● Household groups an essential channel for health and nutrition education and behaviour change.	● At least half of families already changed behaviour in feeding and caring for children and pregnant women; ● Peer-to-peer exchanges crucial for knowledge sharing and behaviour change; ● Families applied new knowledge on nutrition, nutritious crops and school feeding.	● Village health workers gained knowledge on NSA; ● Improved caring and feeding behaviour for children and pregnant women; ● Household groups meetings enhanced social capital.	● Better home cooking practices learnt from the school feeding program; ● Parents spend more money on food for children; ● Parents working away unable to take good care of children.	● Better home cooking practices learnt from micro-entrepreneurs; ● Parents pay more attention to nutrition, feeding their children, and school meals. Some bought food from micro-entrepreneurs for children not at school; ● Micro-entrepreneurs and parents communicate about school meals and food intake.	● More exchange on NSA knowledge and practices among community members and households lead to behaviour change; ● Parents increased knowledge on nutrition and health care. They will continue new behaviours after project ends; ● More social exchange in community; ● Parents working away unable to take good care of children.
HGSF pathway	● Education sector needs multi-stakeholder collaboration to implement school feeding program; ● At least 15% of households sell surplus agricultural products to micro-entrepreneurs for school meals; ● Reduced undernutrition among nursery school children; ● School meals contributed to increased nursery school attendance; ● Children requested same meals at home; ● District Education Committee will continue school meals when program support ends.	● Selection of committed/active micro-entrepreneurs; ● Increased use of home-grown foods in school meals; ● Parents and teachers communicate about school meals and nutrition education; ● Reduced undernutrition among nursery school children; ● School meals contributed to increased nursery school attendance; ● Most parents willing to pay for school meals when program support ends but some are unable to pay.	● Parents pay more attention to nutritious breakfasts for nursery school and younger children; ● Parents bring children to school more regularly and on time; ● School feeding contributed to behaviour change in feeding and caring for children and pregnant women; ● Most parents willing to pay for school meals when project ends but vulnerable households cannot.	● Teachers, parents and micro-entrepreneurs communicate more about nutrition and school meals; ● Reduced undernutrition among nursery school children; ● School meals contributed to increased attendance at nursery schools; ● Children changed hygiene habits and food preferences; ● Increased use of home-grown foods in school meals; ● Micro-entrepreneurs provide alternative payment plans for families that cannot pay on time.	● Local food sources are used for school meals, including micro-entrepreneurs’ own products; ● Children like school meals and look healthy; ● Positive effects of school meals motivate micro-entrepreneurs to maintain good quality; ● Increased trust in the capacity of micro-entrepreneurs; ● Most parents willing to pay for school meals after project ends but some are unable to pay; ● Micro-entrepreneurs provide alternative payment plans for families that cannot pay on time.	● Micro-entrepreneurs and parents communicate about cooking and intake of nutritious foods; ● Children love school meals and expect the same food at home; Reduced undernutrition among nursery school children; ● All want to sustain the school feeding program but vulnerable households are unable to pay the full cost.

## Production–consumption pathway

The main entry point to the production–consumption pathway was the implementation of nutrition-sensitive and context-appropriate food production. Based on program design, implementation of improved agricultural models was enabled through training, agricultural and financial inputs, as well as exchange visits. The agricultural knowledge and skills of the beneficiary farmers increased, as did local availability of nutrient-rich crops and animal food sources. As envisaged in the program theory of change, the adoption of nutrition-sensitive agricultural models contributed to the increased consumption of nutritious foods for the entire household, which led to a perceived improvement of children’s nutritional status. A positive feedback loop resulted from this program component, when successful beneficiaries acted as role models for other households.

### Outcomes

***Increased production of nutrient-rich foods and improved household diets***—Most respondents reported that agricultural knowledge and skills improved, enabling beneficiaries to cultivate their own nutritious food and improve their diets. As stated by a Commune representative: *“The villagers now produce more nutritious food, such as chicken or quail eggs, vegetables, chicken meat. This food is important for the daily intake of children and pregnant women.”* (R6, Commune health). Similarly, villagers reported: *"We noticed a change in the food portions in families: more eggs and vegetables from household-scale planting and animal raising, so the families can save money. We can also supply more food for our families."* (G3, Village FGD). The partial shift from cash crops to nutrient-rich crops was also frequently stated, and observed by MCNV and District staff during monitoring visits. In the words of a District official: *“I think that [now] the local people are more concerned about non-cash crops, for example having eggs or home gardens to have fruit and vegetables they can use to increase nutrient intake for the children and the family.*” (R1, District health).

Respondents also pointed at the general perception that self-produced food was healthier than food purchased from external sources: *“Food raised by ourselves is safer and better for our family’s health.”* (R12, Parent/farmer) and *“In the market now they sell a lot of unhealthy food. We don’t know the origin of this food, and most of it lacks nutrients.”* (R1, District health).

Multiple respondents indicated that people’s access to a variety of locally produced nutritious foods was associated with improved diets, not only of children and pregnant women but of the entire family. The message was reiterated at village, commune and district level: *“We have learned to take better care of our chickens and we can use their eggs to improve our meals, especially for our children.”* (R26, Parent/farmer) and *“Now, many households are also focused on raising chicken for eggs and planting vegetables to improve their own daily intake and also for other households in the community.”* (R3, District agriculture).

***Reduced food expenditures and income generation***—Besides increasing household consumption of nutritious food, adopting improved agricultural models proved to be economically beneficial, by saving food-related expenses. A commune representative explained the double benefit: *“This activity created eggs and vegetables in the community. This will help the villagers to save money for buying food, while giving them nutritious food for their children every day.”* (R5, Commune agriculture). The actual outcome was reported at village level: *“Since project support started, beneficiary households have reduced their spending on buying vegetables. They also buy less eggs from outside to cook porridge because they use eggs from their own chickens.”* (R23, Parent/farmer).

Apart from reducing food expenditures, the agricultural component led to another outcome not envisaged at the outset: income generation. The NSA program monitoring reports show that in 2020, at least 15% of the families produced a food surplus which was sold either to other households or to the micro-entrepreneurs responsible for HGSF procurement, or given for free to other community members, as reported by parents/farmers: *“They [farmer beneficiaries] can give vegetables for free, but for meat and eggs, they will sell 15–20% of the products to other people”* (R26, Parent/farmer) and *“Sometimes I sell chickens to Mrs. Pham (micro-entrepreneur) or other households. I sell few eggs as I often use them to feed my daughters. I do not sell vegetables; we usually eat them or give them to relatives.”* (R10, Parent/farmer).

### Successful elements of program theory

***Improved agricultural knowledge and skills combined with access to inputs***—It was widely stated that local agricultural practices had improved thanks to the acquired knowledge and skills. Beneficiaries were stimulated to implement and invest in agricultural models different from the conventional cash-crops, in combination with the provision of material inputs. Training and exchange visits contributed to improving knowledge. These changes were easily observable during monitoring visits. A District official explained his satisfaction with peer-to-peer learning: *“One very good point of this project is that we create meetings at the households. (…) we bring people together to exchange and share experiences. We also had exposure visits to households who have a very good model, in a village in another commune.”* (R3, District agriculture).

***Context-appropriate agricultural models and program flexibility***—The specific agro-ecological conditions of each village required a tailor-made approach, resulting in a mosaic of models across the five villages. While fish and frog farming were suitable in a few cases, other options, such as chicken raising and vegetable production, were more widely applicable. The variety of models was recorded during monitoring visits by MCNV and District staff. Training was instrumental for joint development of production systems with a good fit-to-context. Another important aspect was the flexibility of the program; agricultural staff had opportunities to test and adjust models in collaboration with beneficiary farmers. According to the MCNV office chief, such flexibility motivated local staff to design models with better fit, instead of conventional top-down blanket solutions: “*When we introduced the model, it was tested, it might fail or it might succeed. The government staff were allowed to do that, unlike the government programs, where they get the design from the province and use a top-down approach to district and commune; they just follow the exact design, even if it doesn’t fit with the reality. But in our program there is really enough space for them to test, adjust and learn. That also motivated the government staff a lot when they joined the interventions.”* (R30, MCNV office chief). This approach was very much appreciated by all participants. Farmers frequently expressed positive reactions to the new models and the program flexibility: *"The project provides support to us to raise small animals, such as chicken for eggs, quail, crickets, earth worms, that are appropriate for our conditions. If we like raising quail or chicken that we think are appropriate for us, then we register to receive support."* (R12, Parent/farmer); *“Now we know how to raise chicken for eggs and plant vegetables for nutritious food so we will continue."* (R10, Parent/farmer); and *“I have more experience because of the project. I had the chance to join trainings and exposure visits to learn. Households in my group said that they liked this project very much.”* (R23, Parent/farmer).

### Facilitators and barriers

Role models were important facilitators in the production–consumption pathway. This social mechanism, common in the local culture, was known to the implementers and successfully integrated in the implementation strategy. The empowering effect of role models and enhanced self-confidence was confirmed across different stakeholder groups. Barriers included the socio-economic conditions of vulnerable households, land and water scarcity, animal diseases, and limited access to animal feed. Details and illustrative quotes are presented in Table 4.

**Table 4:** Overview of facilitators and barriers per pathway

	Production–consumption	Caring and feeding	HGSF
**Facilitators**	***Role models and increased confidence*** ● Households with successful experiences acquired a role model status within their communities; ● Exposure to such positive experiences and their visible benefits gave other beneficiaries the confidence and motivation to implement improved agricultural systems; ● Increased confidence and pride in their achievements were important elements in the successful program implementation. Micro-entrepreneur (R17): *“Since my father started this model [chicken and pig raising] many people have learnt to apply the model like him.”* MCNV office chief (R30): *“People are confident and proud that they achieved what they did. Two years ago, they only focussed on cassava, rice and acacia, and now they can do many things. They feel that they can control it and are proud of it. It is an important aspect.”* Commune agriculture (R5): *“The second most important change is that the community now believes in their capacity to implement the agricultural models; in the past they never raised quails, crickets or earthworms, but now they can. Confidence is very important for them to maintain and develop these models.”*	***Role models and increased confidence*** ● Household group leaders became role models for other parents especially for mothers. They transferred the new knowledge and skills acquired by training, infusing confidence on the results of the improved practices. VHW (R11): *“ I saw mothers join household group meetings regularly […]. The group heads who received training shared with group members.The mothers have made changes because they now understand. For those with children under 5, they learned how to take care of their children, how to improve child hygiene and how to cook the right food for their children. These changes make it better than in the past.”* District health (R1): *“For ethnic minority people it should be quite easy; when they see someone in the community who has success, they are eager to learn. […]. These positive role models are very active in supporting other households to learn from them. […]. So we should focus on role models for others to learn and change their behaviour.”* District education (R2): *“I think the local people see the benefits and their responsibility when they are involved in the project, so they feel confident and responsible when they join the project.”*	***Role models and increased confidence*** ● Parents whose children had visible improvement in nutritional status, became role models, sharing their successful experience with others at school and HGMs; ● Exposure to positive examples and their results enhanced confidence and motivated other beneficiaries to replicate good practices. Commune nursery school principal (R4): *“At school, when a child looks healthy, other parents asked teachers about the parents of that child, because they wanted to meet them.”* ***Change agents*** ● Both nursery school children and micro-entrepreneurs were perceived as agents of change; ● Children’s appreciation of the school meals and their request to eat the same food at home induced changes in parents’ cooking and feeding practices; ● Micro-entrepreneurs engaged with the community not only by supplying school meals but also by selling food and demonstrating recipes to community members; ● Multiple interviewees confirmed that the micro-enterprises also provided benefits to children not registered at nursery schools. VHW (R9): “For mothers of children not yet attending preschool, for both well- and malnourished children, mothers buy nutritional porridge for breakfast.”
**Barriers**	***Socio-economic conditions of vulnerable households*** ● Poor households lacked time to implement the agricultural models. Their need for waged labour far from home was noted as a main constraint; ● Furthermore, economic conditions affected the extent and quality of implementation. Commune agriculture (R5): “*Most households that do not change their behaviour, it’s because they have a very difficult economic situation. To earn money they have to go to work far away; they may work away from the community for 10 or even 20 days.”* District agriculture (R3): *“Some households implemented the model not very well; they could not expand the number of their chickens, or had fewer chickens. I think that this is all related to the economic situation of the household.”* ***Land and water scarcity*** ● Despite having acquired sufficient skills, a large number of beneficiaries were unable to sustain their vegetable production during the dry season due to lack of water; ● The change of agricultural practices was also constrained by the lack of land, which limited the scale of production; ● Farmers recognised a lack of drought-resistant crops, such as pumpkin, bitter gourd and papaya. Phu Giang Village FGD (G3): *“We lack water in the dry season. We have to fetch water from places away from home, but not enough for watering vegetables.”* ***Animal diseases and limited feed*** ● Beneficiaries received chickens to produce eggs but faced several challenges, including animal diseases; ● Building on the lessons learnt from implementation, the NSA program trained beneficiaries on techniques, such as corralling and vaccinations, to minimize the spread of diseases. The applied strategies were not successful enough; ● Early on, shortages of animal feed were perceived as a barrier. After the introduction of alternative feeding systems with crickets and earthworms, that barrier was overcome. District agriculture (R3): *“Chicken diseases are still happening, even though we provided vaccinations.”*	***Socio-economic conditions of vulnerable households*** ● Barriers to change were found among the most disadvantaged households, which tend to have more children but less time for their care; ● Management of their limited financial resources constrained their ability to change; ● Some families find caretakers for their children but inadequate caring and feeding practices may not change if those caretakers are not involved in the learning activities. VHW (R11): *“Households who earn money and spend it within a day have a very difficult situation. It is a challenge to convince these households because their economic situation is too hard.”* ***Household priorities and lack of time*** ● In spite of the positive attributes of HGMs, participants’ priorities and/or lack of time proved to be a constraint; ● Lower engagement was partly attributed to communication materials being insufficiently tailored to the illiterate participants, hampering uptake of complex messages, such as vaccination and seasonal diseases. VHW (R28): *“If I or other group leaders ask, they come, but I must talk strictly; if I suggest gently to have a meeting about their children, they said they don't have time, they are tired, they work every day and don't have time for group meetings.”*	***Socio-economic conditions of vulnerable households*** ● The school meal subsidy provided by the NSA program was crucial at the beginning, as it encouraged community buy-in. However, as the program progressed, the costs raised issues regarding sustainability; ● Limited financial capacity to contribute became a significant constraining factor especially for the poorest households, in spite of their willingness to continue. Village teacher (R8): *“Actually, parents having better economic status will support this model. However, with parents having low income, it's a challenge for them.”* Micro-entrepreneur (R14): *“I think most parents like the school meal, they are happy when their children are fed with good and tasty food, but for some families it is not easy to pay for 100% of the school meal.”* Parent/farmer (R12): *“For my group, two other members have chickens and have children at pre-school. They are willing to pay for the school meal but for other households in the group, maybe 50% are willing to do that.”*

## Caring and feeding pathway

In this pathway, the monthly HGMs were instrumental for increasing parents’ awareness and knowledge of caring and feeding practices. In turn, enhanced knowledge was associated with positive changes in cooking, breastfeeding and WASH practices, and in healthcare-seeking behaviour. As envisaged in the program theory of change, the adoption of enhanced caring and feeding practices contributed to perceived improvements in CU5 food intake and health status. Similarly to the production–consumption pathway, role models were crucial in the replication of best practices by others.

### Outcomes

***Improved feeding practices***—Most respondents acknowledged the positive changes in feeding practices and established a clear link between the knowledge acquired in the HGMs and children’s improved diets. Breastfeeding practices improved, with many mothers maintaining exclusive breastfeeding for the first six months. Furthermore, parents realized the importance of providing children with a nutritious breakfast before going to school: *“Before, parents fed breakfast to their child with anything, not necessarily nutritious food. For example, instant noodles, or even no breakfast, but now the parents care about nutritious breakfasts like nutritious porridge.”* (G2, Commune FGD). Thanks to the cooking demonstrations, mothers improved their cooking skills and started preparing nutritious meals for the entire family, often using home-grown products. One mother described the effect of new knowledge on her child feeding practices: *“I didn’t follow any menu when feeding my children. I just fed them with whatever I had. Since the project, we take better care of our children. We had no idea about malnutrition but thanks to this project, we gained knowledge.”* (R15, Parent).

***Improved caring practices***—Better knowledge motivated parents to improve their caring practices, especially as a result of health promotion activities. Implementers noticed that the vaccination rate increased and parents were better able to recognize common children’s illnesses and sought health care. Furthermore, increased attention to WASH practices and a healthier living environment were observed during monitoring visits, and were explained in the commune FGD: *“I recognize that parents are proactive in personal hygiene for their children even when the children are not ill. They help their children brush their teeth, wash faces and hands in the mornings, and put on clean clothes. They also clean their house, clean animal cages and fence the garden to avoid disease transmission.”* (G2, Commune FGD).

### Successful elements of program theory

***Increased knowledge on child caring and feeding***—As in the production–consumption pathway, knowledge was a key element of success. Improved practices were strongly linked to the increased awareness, knowledge and skills that parents gained in the HGMs. A VHW illustrated the link between knowledge, children’s nutritional status and the role of HGMs: *“Knowledge is necessary, that helps children gain weight. Mothers who lacked knowledge and awareness now join household groups, gain knowledge and take better care of their children.”* (R9, VHW). Nutrition counselling was rarely mentioned by respondents at village level. It was, however, said during the district and commune FGDs to have played an important role in knowledge creation; parents seemed to follow recommendations from external authorities, such as province or district doctors.

***Motivation from observed benefits***—The implementers’ hypothesis, that positive outcomes for the participating beneficiaries would convince the remainder to change, proved correct. A District official commented: *“For ethnic minority people it should be quite easy; when they see someone in the community who has success, they are eager to learn.”* (R1, District health). Once a critical mass of beneficiaries adopted the improved feeding and caring practices and saw positive results, others were motivated to replicate the good practices. The most convincing evidence supporting behaviour change was the improved nutritional status of their children. During the HGMs, children were weighed and their growth monitored. Parents could observe tangible results, take pride in their achievements or be motivated by improvements in other children.

***Skilled household heads and sharing opportunities***—Active household heads, capable of organizing monthly group meetings and delivering complex messages, were perceived as strong facilitators of well-functioning household groups. A District official explained: *“When we select the household group heads, we prioritize young women who finished at least secondary school. Because this group are young and have knowledge, it is easier for them to attend and to learn from the health staff, and they can more easily transfer that knowledge to the household group members.”* (R1, District health). Linked to this, HGMs were acknowledged as a new and effective platform for knowledge exchange. Different stakeholders confirmed that parents liked the HGMs because of the topics covered, participatory cooking sessions, and peer-to-peer learning. Participation in such meetings during monitoring visits by MCNV and District staff confirmed the increased exchange and communication reported by other stakeholders. All of which, helped to build social capital.

### Facilitators and barriers

Household group meetings provided the forum in which the group leaders who were trained became role models for other parents especially for mothers. The socio-economic conditions of vulnerable households were again a key barrier along with household priorities and lack of time. Illustrative quotes for the barriers and facilitators of this pathway are presented in Table 4.

## HGSF pathway

School meals, implemented with trained micro-entrepreneurs and teachers, were the key entry point to the HGSF pathway. As envisaged, the provision of nutritious meals had a positive effect on children’s diets and on school attendance. An unanticipated spin-off was that, on children’s request, parents were motivated to prepare similar food at home. The visible improvement in children’s nutritional status was the strongest incentive for parents to support the school meals and change their home cooking practices.

### Outcomes

***Improved dietary intake and reduced children’s undernutrition***—As reported in Box 1, before the NSA program, there were no school meals in any of the villages in Phu Mo commune. Nursery school children were often not adequately fed as their parents worked in the fields throughout the day. All stakeholder groups agreed that, compared to baseline, the nutrient-rich school meals and the related adoption of better feeding practices greatly contributed to improved dietary intake of the nursery school children. A micro-entrepreneur explained: *“At school children eat more than at home [….]. At lunchtime, they usually eat two bowls of rice, one with the main dish consisting of meat or fish and one with nutritious soup. This is how children are fed at school.”* (R14, Micro-entrepreneur).

All stakeholder groups mentioned the better nutritional status of the nursery school children. Several sources reported a perceived decrease in undernutrition; a micro-entrepreneur commented: *“Previously, the children looked thinner and shorter but now they look much much better.* (R13, Micro-entrepreneur). From a teacher: *“The malnutrition situation has improved outstandingly. Before this model was implemented, the malnutrition rate was significantly higher.”* (R8, Teacher). Many parents noticed the improvement and felt more motivated to support school feeding: *“I think it’s clean food and has enough nutrition for malnourished children…it helps to improve the children’s health. We are very happy about that.”* (R12, Parent/farmer). The perceived reduction in children’s undernutrition was indicatively confirmed by the anthropometric data collected routinely, three times a year, by the teachers according to the Ministry of Education and Training protocol. In 2017–2018, there was only a slight difference in the proportion of underweight children between the start and the end of the school year (from 24% down to 22%); to be noted that the school meal program started as a pilot only in two schools just before the last growth measurement of that academic year, so no effect could be observed. In 2018–2019, when the full implementation of the school meal program started, the reduction in the aggregated underweight was noticeable (from 26% to 14%). Finally, in 2019–2020, a reduction in underweight from 33% to 17% was already observed in the first quarter (September–December), before children had to stay home during the COVID-19 quarantine.

***Home cooking and spending practices***—Important behaviour changes were also observed in home practices. Seeing their children become healthier motivated parents to improve their home cooking and their spending habits. As a District official explained: *“Parents start to be concerned about food intake, because through the school meal the parents see and recognize that the children really like the new foods and they try to learn and make the same new dishes at home.”* (R2, District education). Parents were also more likely to buy nutritious food at the micro-entrepreneurs’ shops, prioritizing food over other expenditures, as an official said: *“There is a change, for example, when the family has cash, they now have the priority to buy milk and nutrient-rich porridge instead of only buying beer or cigarettes.”* (R1, District health).

***Increased school attendance and parents’ spare time***—School meals were also associated with increased attendance to school. Parents made sure children were on time and started to accompany them to school, as reported by several respondents: *"Children changed considerably since this model has been applied. They go to school on time and more regularly."* (R8, Teacher) and *“In the past, children went to school by themselves, but now parents, especially mothers, go to school with their children*.” (G1, District FGD). These qualitative findings aligned with secondary data obtained from the Ministry of Education and the principal of Phu Mo nursery schools. According to these sources, there had been a slight increase in the number of students registered in the school year 2019–2020 (132 children) compared to the previous two years (118 in 2017–2018; 116 in 2018–2019). Furthermore, an increase in the average attendance ratio had also been recorded (88% in 2017–2018; 97.6% in 2018–2019; and 98.6% in 2019–2020), with children staying at home when ill. The increased school attendance and the fact that children would eat at school had an additional positive spin-off for the parents, leaving them more time for farming and other activities.

***Improved social capital***—Implementers often mentioned that, because of regular meetings involving micro-entrepreneurs, teachers and parents, their social capital increased, which was perceived to be a result of the adoption of school meals and related feeding practices. Stronger bonds and trust, improved communication and knowledge exchange about children’s health, the creation of an informal community of practice engaging in healthy and nutrition-sensitive behaviours, were all part of a process that expanded the range of possibilities to address undernutrition as a community rather than as individuals. This increased cohesion and communication in the community was observed by MCNV and District staff who visited regularly. A District education staff described the cycle that enhanced social capital: “*One positive impact I see is the collaboration and relationships among the community. For example, through the school meals or through selling breakfast or porridge, the children now have better food intake, and look more healthy and cheerful. When they come home, they praise the food, which makes parents believe in or even love the teachers and local microenterprise.”* (R2, District education).

### Successful elements of program theory

***Motivation from observed benefits***—As seen in the caring and feeding pathway, visible positive results were an important element of success. Child growth monitoring during the HGMs increased parents’ awareness of the benefits of the school meals. A mother reported: *“The child gains more weight when she joins the school meal, I think because they have good food intake and drink milk. In the past, she was 11 kg; since she joined school meals, I weighed her at 13 kg.”* (R10, Parent/farmer).

***Eating together***—Eating at school together with other children contributed to the success of the HGSF. Teachers encouraged children to eat, as reported by a VHW: *“It's not only because of the good food but there are many children and they like eating, they look at each other and eat and Mrs X encourages them to eat so the children gain weight.”* (R28, VHW).

### Facilitators and barriers

The positive examples of successful role models contributed to enhance the confidence and motivation of other beneficiaries to replicate the good child feeding practices observed in schools. Both nursery school children and micro-entrepreneurs were perceived as agents of change. The main barrier was the low socio-economic condition of vulnerable households. Additional information and illustrative quotes can be found in Table 4.

## Synergies, successful elements of program theory and facilitators across pathways

A reinforcing relationship was observed between the caring and feeding and the production–consumption pathways. Thanks to the improved agricultural models, availability of local nutrient-rich foods increased. This allowed parents and other community members to apply the knowledge acquired in the HGMs to change their feeding practices. The synergy was strengthened during the monthly meetings, when participants were exposed to nutrition information as well as promotion of nutrition-sensitive agriculture systems. Furthermore, home-grown food was provided for cooking demonstrations. These links between nutrition and agricultural training worked well. They were particularly meaningful for beneficiaries involved in both intervention components, but also for others. As explained by a Commune health representative: *“When we organize a cooking class and guide the villagers to prepare nutritious food, we often advise the household to use chicken or quail eggs or vegetables, which are available thanks to the support in the agricultural sector.”* (R6, Commune health).

Another important synergy with high potential was observed between the production–consumption and the HGSF pathways. While the former contributed to an increased supply of locally produced nutrient-rich fruits, vegetables and animal foods, the latter created local demand for such foods, thus reinforcing the “home-grown” dimension of the intervention. The fact that micro-entrepreneurs bought the production surplus of local beneficiary farmers further incentivized them to implement the new agricultural models. Although the local production surplus played a modest role in the HGSF, it showed potential, as one micro-entrepreneur said: *“Rice is nearly 100% locally supplied. Around 30%-40% of chickens are bought from local people. About 20% of eggs are locally supplied. 50% of vegetables are provided by locals and mobile vendors.”* (R7, Micro-entrepreneur).

An interesting spin-off of the production–consumption pathway was the development of an income sub-pathway selling surplus products in the community and to the micro-entrepreneurs for the HGSF. This unanticipated outcome is explained by the flexibility of the program strategy. While the program actively promoted household consumption of eggs and home garden produce, it did not discourage selling any surplus to generate income. The program strategy thereby contributed to developing an income sub-pathway, as well as strengthening the HGSF pathway.

An important element of success overarching all three impact pathways related to the role and approach of the implementing NGO. MCNV was instrumental in the facilitation and coordination of the stakeholders at multiple levels. At the local level, MCNV worked closely with the district government and staff from the agriculture, health and education sectors, further building on the long-term partnership developed with Dong Xuan authorities since 2008. MCNV did not aim to set up *ad-hoc* program structures or make radical changes in existing structures, but engaged local institutions in a shared vision of change. Working with existing structures is part of MCNV’s time-tested approach to sustainability: increasing local actors’ understanding of the persistent undernutrition problem and its determinants, addressing the problem with actions appropriate to local context and potential, and integrating existing human and financial resources, as well as policy-related opportunities, into program design. MCNV supported the strengthening of local capacity and stimulated participation, empowerment and ownership of the local actors through co-design of interventions, trial and error, co-funding, autonomy in decision-making and authorized implementation. At the national level, MCNV liaised with strategic actors, such as the National Government, the Scaling Up Nutrition (SUN) alliance for Vietnam, and the National Nutrition Institute, to influence the nutrition security agenda and foster scaling-up. MCNV collected proof-of-principle throughout the NSA program and used reflexive cycles of monitoring and evaluation, involving the local team and the scientific partners, to adapt and progressively improve the program. The acquired evidence base was valuable not only for implementation but also for planning scale-up. As an NGO, MCNV has more flexibility to test and support pilot models difficult to finance with public funding. Once a model proves successful, the public sector can adapt and integrate it into official sector plans. *“The other thing is that the MCNV is flexible in project implementation, when we had any issue or recommendation, there was a good collaboration between the MCNV and the sector to adjust or improve implementation.”* (R1, District health).

Finally, it should be noted that in addition to pathway-specific facilitators, other factors external to the program, such as improved roads and Internet access, also contributed to an enabling environment for the overall implementation of the NSA interventions. According to a District official: *“Currently, villagers also have access to smartphones and Internet, so besides learning knowledge from the sector, they also learn through the Internet. This may help them to have better behavioural change and better implementation of the agricultural nutrition model.”* (R1, District health).

## Sustainability prospects

### Opportunities

***Community empowerment***—Most respondents suggested that the sustainability of the NSA interventions depended on capacity built at local level. The acquired knowledge and skills empowered the communities and gave them confidence in their capacity to continue what the program started, as stated by the Commune school principal: *“This project will create sustainability, because at this moment we have improved the nutrition status and the awareness of the parents has already changed, so even if the project stops, the parents will continue because of their changed awareness.”* (R4, Commune education).

***Observed benefits and commitment***—Common to all three pathways was the ‘self-sustaining’ loop stimulated by the perceived benefits of the NSA interventions. Visible results were a catalyst of change for early adopters and a source of inspiration for others. A District official emphasized the benefit-driven motivation to sustain the improved agricultural models: *“I think that people will continue to sustain this model because now they are able to create breeding stock and seedlings, and they are aware of the benefits of this model. In addition, I think this project created new behaviour, especially new awareness on preparing nutritious food for children. This will create good results in health, so I think people will sustain this behaviour.”* (R3, District agriculture). The observed benefits also resulted in strong support from local authorities to continue and even scale-up the NSA approach as stated by a District official: *“I think we can scale up the NSA approach in the district. In 2020, the district and MCNV agreed to plan the scale up to all the upland villages of Dong Xuan district. In 2020, the agricultural department has already allocated quite a lot of its own budget for the agriculture-nutrition model.”* (R3, District agriculture).

### Challenges to sustainability

***Limited capacity of local implementers***—The lack of skilled local human resources for implementation and maintenance of activities was often mentioned as a limit to sustainability. A critical mass of local implementers with sufficient capacity was deemed crucial, as they will retain the acquired knowledge and skills in the commune and because the Government relies on them to support the communities. The challenges included the remoteness of these villages and the difficulty of finding and training local people to facilitate activities and deliver complex messages.

***Reliance on NGO support***—Throughout the NSA program, the implementing NGO (MCNV) played a central role in the horizontal and vertical coordination of the activities as well as providing technical and modest financial support. Several respondents voiced the potential risk that at the end of the program, a void could appear that would be difficult to fill, as a District official explained: *“So far, MCNV played the role of coordinator among the sectors. When the project ends, maybe we will have difficulties with who will play that role. So far MCNV also supported financially, so when the project is finished, maybe it will be difficult to find financial resources.”* (R3, District agriculture). The expectation was that the district government would take over both the coordinating and supporting roles to sustain the collaboration.

***Financial constraints of school meals***—Key to the HGSF was the downscaling subsidization scheme; parents were planned to fully cover the costs of school meals from 2021. Most respondents agreed that while the majority of the households, motivated by the good results, could be willing and able to support school meals, the most disadvantaged households, potentially those who most need it, would struggle and eventually drop out.

***Low resilience of local systems to external shocks***—The COVID-19 pandemic not only halted all program activities but also accentuated the limited resilience of local (food) systems to external shocks. The negative consequences became visible within a few months after the quarantine, with children having lost most of the weight gained since they started the school meal program, as testified by a teacher: *“After the pandemic period, when children had stayed at home, they lost weight and went back to their original weight.”* (R18, Teacher).

### Solutions for sustainability

***Self-maintenance of school meals***—Respondents envisioned a number of alternative solutions, such as customized payments or fund mobilization, to sustain the school feeding after the end of the NSA program, as discussed in the district FGD: *“First we will continue to convince the parents to maintain the school meal; second we will discuss alternative payments with the local micro-enterprise, for example, when the local micro-enterprise hires labour to harvest cassava or rice, they can hire these households. Or they can wait until these families harvest their crops or earn money and they can pay then”.* (G1, District FGD).

***Continued support for more vulnerable households***—The most disadvantaged households could not benefit from the interventions, except for the school meals, for which the flexibility of teachers and micro-entrepreneurs in case of delayed payments was instrumental in continued support. Respondents recommended to continue supporting them, as described by a commune health worker: *“There are households who did not change, who did not pay attention to the health or nutrition of children. (…) I think we should have a solution for these difficulties. For example, we continue to promote behavioural change. We should create a good model which people can learn from and follow. For the households with difficult economic situations, when they have a risk like chicken diseases, we should continue to support them.”* (R6, Commune health).

## Discussion

While much research has been conducted on the impacts and pathways of NSA programs, there is still limited evidence about the processes and lessons learnt from the implementation of such programs. Our study contributes to the growing body of literature of qualitative and mixed-methods studies focusing on process assessment of NSA programs by deepening and enriching the insights of NSA implementation research and impact evaluations on the why and how changes occur [27, 32, 52, 53, 56, 65; among others]. This qualitative assessment, in particular, was conducted to improve the implementation and scale-up of the current NSA program, but also to share lessons with practitioners and researchers in the field. Insights from this study could contribute to the design of future NSA programs.

Our results show that, overall, the envisaged impact pathways functioned as intended and that several promising unplanned synergies emerged during implementation. Positive outcomes were observed in relation to children’s and households’ diets, feeding and caring practices, school attendance, increased social capital, and income generation. Some outcomes were pathway-specific, others resulted from the interplay among pathways. While program flexibility in the implementation of context-appropriate agricultural models was an important element of success in the production–consumption pathway, benefit-driven motivation and improved knowledge triggered positive changes in all pathways. Change agents, role models and increased confidence were perceived as key facilitators in the process. However, the socio-economic conditions of poor households were a critical barrier to their participation as well as a sustainability challenge. Community empowerment and the recognition of the NSA interventions’ positive outcomes could play a key role in sustainability. However, challenges related to the low resilience of local systems, the limited capacity of local implementers, and the dependence on external support need careful attention.

**Improved knowledge** among beneficiaries was a common element in all three pathways in our study, but was perceived as **a critical factor for behaviour change especially in the production–consumption and caring-feeding pathways**. The empowering effect of knowledge and its contribution to improvement of beneficiaries’ behaviours and practices is recognised in the literature [34–38]. Such an effect is potentially greater when beneficiaries receive both agriculture- and nutrition-related knowledge; the combination allows them to apply their learning on nutrition starting from production of their own nutritious foods [36, 39, 40]. In fact, nutrition education alone can improve child nutrition only if access to food is not a limiting factor [41], while distributing inputs and providing basic agricultural support may not be sufficient to increase children’s consumption of nutritious food if nutrition education is lacking [42]. However, when the complexity of programs increases, information overload may pose a challenge. Nordhagen et al. [27] raised doubts about *“whether it is realistic to expect a targeted participant in an NSA program to simultaneously absorb new information across a wide set of domains (e.g., agriculture, WASH and gender norms) and to make changes concomitantly on multiple fronts”*, especially within the short timeframe of most projects. In our project, training and exchange visits using a participatory approach and the stimulation of peer-to-peer learning were instrumental to sharpen beneficiaries’ knowledge and skills, which in turn led to changes in caring, feeding and agricultural practices. Roche et al. [43] suggested periodic refresher trainings to ensure knowledge is not forgotten and is actively applied. Furthermore, not only may it take time for knowledge to be adopted [44, 45], but also training on its own may not be sufficient to induce actual changes, given the complex interaction between motivational factors (psychological, cultural, social and economic) and the processing of information [46]. In our group, HGMs were particularly valued for being an effective and practical platform for knowledge exchange, with hands-on activities and exposure to good role models; they also expanded the range of knowledge-sharing opportunities. The importance of practical training, demonstrations and group sessions for knowledge sharing and interactive learning has been highlighted [38, 47, 48]. However, the fact that training materials used here were insufficiently tailored to the illiterate participants hampered the uptake of complex messages and raised the need for communication materials with more visual aids, as reported elsewhere [32].

Our study clearly showed how **the tangible benefits of NSA interventions were decisive in motivating the uptake and replication of improved practices**. In line with findings of Okello et al. [48], exposure to peer role models and their success stories boosted beneficiaries’ motivation and confidence in their capacity. Our study also validated the idea that integrating known culturally appropriate social mechanisms in the implementation strategy can be effective to stimulate spontaneous horizontal spread and enhance the self-sustainability of the interventions. The role of benefit-driven motivation in adoption is acknowledged in the NSA literature. Several articles refer to motivational factors, such as better nutrition and health, enhanced coping mechanisms for food and nutrition security, empowerment, comparative advantages of promoted crops, and income opportunities [47, 49–51]. However, while we found that the improved nutritional status of children was a key catalyst, others remarked that nutrition and health benefits may not be sufficient drivers for adoption [52]. Such benefits should be combined with more enduring motivators, such as income and empowerment, for NSA interventions to be adopted and sustained.

**Program flexibility, built into the intervention design, was instrumental in the development of context-appropriate agricultural models**, which, in turn, stimulated adoption. The importance of program flexibility in addressing design limitations based on lessons learnt from implementation and monitoring is documented in several studies on NSA [36, 57, 70; among others]. However, only a few studies present flexibility as a built-in feature of the program [27, 53–55]. According to Nordhagen et al. [27], in the context of complex programs, such as NSA, the use of lessons learnt to improve interventions is critical, but real-time learning is possible *“only if there is sufficient flexibility in the workplan, budget and staffing”*. Furthermore, Nielsen et al. [53] and Talukder et al. [55] highlighted the close relationship between program flexibility and a functional partnership, which is instrumental for timely and successful adaptation of the program, as observed in our case. Finally, Cole et al. [54] and Olney et al. [32] remarked on the usefulness of a program theory of change in enhancing program flexibility by flagging bottlenecks and enabling identification of suitable solutions together with relevant stakeholders. The NSA program documented in this study could have benefited from that approach (for instance, making explicit the already known land and water scarcity problems), had the program theory been developed at the onset. Not only would this approach have strengthened the design of the NSA program, but it would also have made decisions concerning the internalization of contextual factors in the program strategy, such as role models and change agents, more consistent as intervention design factors.

We found that **community empowerment grounded on the capacity built at local level by the NSA program** **was perceived as an opportunity for sustainability**—the acquired knowledge and skills having increased communities’ confidence to continue what the program started. This finding is not uncommon in the NSA literature, with numerous articles stressing the importance of community empowerment and ownership to enhance sustainability of NSA programs [37, 44; among others]. The increased empowerment and ownership are often attributed to participatory approaches and the involvement of communities from early stages [38, 57–59]. Other studies refer to the mobilization of community members as resource persons, such as trainers, in program implementation as a viable strategy towards community empowerment and ownership [36, 51]. Cost-sharing mechanisms and forms of co-contribution are often advocated to increase ownership and sustainability [27, 38, 43, 60]. However, as demonstrated in our study, such mechanisms may penalize the most disadvantaged households.

Furthermore, our study results emphasise the importance of **working with existing local structures** to enhance the fit-to-context and acceptability of the interventions as well as their sustainability and scale-up. The strong commitment and the enthusiasm of local staff participating in the program was the outcome of the trust established by MCNV through many years of working in Dong Xuan district, making it possible to mobilise stakeholders in new communes and villages. While MCNV provided the initial impetus to the program and played a central role in coordination, in practice, much of the program implementation and monitoring was entrusted to local staff. Valorization and strengthening of local structures has been presented as an actual or potential strategy towards sustainability in several NSA studies. Reference is made to the integration of interventions in existing agriculture and health systems [35, 38, 41, 61, 62], with close involvement of local authorities/institutions [37, 59, 63], and the importance of building their capacity**.** Doubts have been raised about long-term sustainability when a central coordinating role is given to a new structure created *ad-hoc* by a project [64].

Our study brought to the forefront a dilemma faced by many programs: **community empowerment/ strengthening existing structures versus reliance on program support**. As voiced by several respondents in our study, the central role MCNV played in coordinating and financing activities was pivotal to the success of the program but may have created a void that is difficult to fill. Even in well-designed programs, there is a concrete risk that certain activities will be discontinued once the project support decreases or ends [32, 36, 56]. To minimize this risk, Haselow et al. [59] recommend that programs have clear entry and exit strategies, with a key role for participatory approaches in the entry strategy for community buy-in and ownership, while capacity building of local resources paves the way to program exit.

NSA is often proposed as a solution to food insecurity and nutrition problems of poor and vulnerable communities. Evidence from a systematic review confirmed that poverty remains a serious obstacle to effective participation in NSA projects [26]. This NSA program in Vietnam was successful in stimulating behavioural change and adoption of improved care, feeding and production practices among beneficiaries with better starting conditions. However, it **failed to benefit the most disadvantaged households who were potentially most in need**. Poverty, lack of time, reliance on external waged labour, household size, and illiteracy were serious constraints to their involvement. Beneficiaries’ financial constraints, workload and lack of time are common factors limiting the adoption of new practices in low-resource settings [32, 36, 65]. For instance, Roche et al. [43] and Sako et al. [38] remarked how difficult it was for poor rural households, who were food insecure and most affected by adverse agro-climatic conditions, to participate in a grain bank program that expected in-kind and financial co-contribution. Another hindrance to behaviour change among more disadvantaged households could result from the ambitious expectation of NSA programs regarding the capacity of beneficiaries’ to absorb knowledge on several topics and make changes on multiple fronts simultaneously, within the relatively short timeframe of a project [27]. In the same article, poverty is noted to undermine people’s absorptive cognitive capacity. In such situations, aggravated by illiteracy as in our case, an excess of choices could be overwhelming and perhaps discourage people from making choices. According to Doocy et al. [66], in post-conflict countries afflicted by population displacement and severe food insecurity, targeting the most vulnerable and poorest households may not be feasible if interventions are not intensive enough to support them. This point may be applicable to very poor and vulnerable ethnic minority households in remote areas, which have benefited less from national socio-economic development. Among the early studies advocating the strengthening of beneficiaries’ capacity, to enhance sustainability of NSA programs, Ayele and Peacock [67] emphasized the positive long-term effects of building capacity of poor rural households, through asset creation and adult literacy and numeracy trainings. Such recommendations are still valid. For NSA projects to be effective, integration with other interventions aimed at improving livelihood and income opportunities for the very poor should be envisaged [68]. Furthermore, as Ali et al. [69] reported, using data from Nepal, growth policies that improve the income of the very poor and protect them from income shocks would be likely to result in nutritional benefits.

However, in the context of Vietnam, historical barriers related to culture, language and trust undermine the ability or willingness of ethnic minority groups to engage in government development programs [11], leaving this responsibility to NGOs. Such challenges align with the on-going debate regarding the scope and complexity of NSA interventions [27] and the co-location by design of NSA interventions with relevant local (development) programs to maximize their synergetic effects [24].

### Strengths and limitations

The participation of respondents who had been directly involved in the NSA program from the start and were familiar with all its components enriched the results of the study, as they had an accurate understanding of the program elements, what worked and what did not, and for whom. Furthermore, MCNV provided an enabling environment for the study thanks to its context knowledge, long-term experience with participatory approaches, and long relationship with Dong Xuan district. Their mobilization of a trustworthy network of contacts at multiple administrative levels was instrumental to continuing the study under COVID-19 restrictions.

Limitations are intrinsic to horizontal content analyses, for instance decontextualization/ overemphasis of quotes. To address them and to ensure coding fidelity, SSIs and FGDs were initially coded by one researcher, with the others involved in the review of raw data, codes and preliminary findings. Measures were also taken to address translation bias. Data collection tools were validated for cultural sensitivity by trained local staff and the same translators were involved throughout the study to ensure consistency and quality of transcripts. Social response bias was minimized by using both SSIs and FGDs. However, there is a risk that individuals may not have authentically shared their experiences, particularly if they did not conform with socially accepted views. Finally, due to COVID-19 restrictions, some interviews had to be conducted remotely. However, thanks to mobile technology and the facilitation by Dong Xuan district staff, data collection could continue with minimal delays.

## Conclusions

Our findings provide empirical support for the important role NSA programs may have in addressing undernutrition, particularly in remote areas, as they can enhance community self-reliance. However, our findings underlined the need for mechanisms and strategies that are able to benefit the poorest and most vulnerable households. In our study, the integration of behaviour change activities to promote improved food production and care and feeding practices in the NSA intervention package proved to be an important strategy to enhance the impact of agricultural innovations on nutrition outcomes. Key aspects included knowledge gain and exchange, increasing social capital, visibility of benefits, flexibility in program implementation, working with local structures, and integration of contextual facilitating factors, such as role models and change agents, in the program design. Though outcomes and influencing factors are very context-dependent, lessons on what worked and what did not work could inform the design and implementation of effective behaviour change strategies in future NSA programs in Vietnam and elsewhere.

## Data Availability

All data generated or analysed during this study are included in this published article. The complete data set is in Vietnamese but all transcribed material can be made available by the corresponding author on request.

## Abbreviations

CU5: Children under 5 years of age
FGD: Focus group discussion
HGSF: Home-grown school feeding
HGM: Household group meeting
MCNV: Medical Committee Netherlands-Vietnam
NSA: Nutrition-sensitive agriculture
SSI: Semi-structured interview
USD: United States dollar
VHW: Village health worker
WASH: Water, sanitation and hygiene

## References

[1] FAO/IFAD/UNICEF/WFP/WHO. The State of Food Security and Nutrition in the World 2019. Safeguarding against economic slowdowns and downturns. FAO. 2019. https://www.unicef.org/media/55921/file/SOFI-2019-full-report.pdf. Accessed 12 Jan 2021.

[2] IFPRI. Global Nutrition Report 2014: Actions and Accountability to Accelerate the World’s Progress on Nutrition. IFPRI. 2014. https://www.ifpri.org/publication/global-nutrition-report-2014-actions-and-accountability-accelerate-worlds-progress. Accessed 12 Jan 2021.

[3] UNICEF/WHO/WB Group. Levels and trends in child malnutrition: key findings of the 2019 Edition of the Joint Child Malnutrition Estimates. WHO. 2019. https://reliefweb.int/sites/reliefweb.int/files/resources/Joint-malnutrition-estimates-April-2019.pdf. Accessed 12 Jan 2021.

[4] Black RE, Victora CG, Walker SP, Bhutta ZA, Christian P, De Onis M (2013). Maternal and child undernutrition and overweight in low-income and middle-income countries. Lancet.

[5] Stevens GA, Bennett JE, Hennocq Q, Lu Y, De-Regil LM, Rogers L (2015). Trends and mortality effects of vitamin A deficiency in children in 138 low-income and middle-income countries between 1991 and 2013: a pooled analysis of population-based surveys. Lancet Glob Heal.

[6] Caulfield LE, de Onis M, Blössner M, Black RE (2004). Undernutrition as an underlying cause of child deaths associated with diarrhea, pneumonia, malaria, and measles. Am J Clin Nutr.

[7] Grantham-McGregor S, Cheung YB, Cueto S, Glewwe P, Richter L, Strupp B (2007). Developmental potential in the first 5 years for children in developing countries. Lancet.

[8] Hoddinott J, Behrman JR, Maluccio JA, Melgar P, Quisumbing AR, Ramirez-Zea M (2013). Adult consequences of growth failure in early childhood. Am J Clin Nutr.

[9] Stewart CP, Iannotti L, Dewey KG, Michaelsen KF, Onyango AW (2013). Contextualising complementary feeding in a broader framework for stunting prevention. Matern Child Nutr.

[10] Khor GL (2008). Food-based approaches to combat the double burden among the poor: challenges in the Asian context. Asia Pac J Clin Nutr.

[11] Mbuya NVN, Atwood SJ, Huynh PN. Persistent malnutrition in ethnic minority communities of Vietnam: issues and options for policy and interventions. International Development in Focus. World Bank. 2019. http://documents1.worldbank.org/curated/en/369601561716089327/pdf/Persistent-Malnutrition-in-Ethnic-Minority-Communities-of-Vietnam-Issues-and-Options-for-Policy-and-Interventions.pdf. Accessed 12 Jan 2021.

[12] Kozel V. Well begun but not yet done: progress and emerging challenges for poverty reduction in Vietnam. Equity and Development. World Bank. 2014. https://openknowledge.worldbank.org/handle/10986/20074. Accessed 12 Jan 2021.

[13] FAO. Mapping the vulnerability of mountain peoples to food insecurity. FAO. 2015. http://www.fao.org/3/a-i5175e.pdf. Accessed 12 January 2021.

[14] Bhutta ZA, Das JK, Rizvi A, Gaffey MF, Walker N, Horton S (2013). Evidence-based interventions for improvement of maternal and child nutrition: What can be done and at what cost?. Lancet.

[15] Ruel MT, Alderman H (2013). Nutrition-sensitive interventions and programmes: How can they help to accelerate progress in improving maternal and child nutrition?. Lancet.

[16] World Bank. Improving nutrition through multisectoral approaches. World Bank. 2013. http://documents.worldbank.org/curated/en/625661468329649726/pdf/75102-REVISED-PUBLIC-MultisectoralApproachestoNutrition.pdf. Accessed 12 Jan 2021.

[17] Balz AG, Heil EA, Jordan I (2015). Nutrition-sensitive agriculture: new term or new concept?. Agric Food Secur.

[18] Herforth A, Harris J. Understanding and applying primary pathways and principles. Brief #1. Improving Nutrition through Agriculture Technical Brief Series. Arlington, VA: USAID/Strengthening Partnerships, Results, and Innovations in Nutrition Globally (SPRING) Project. 2014. https://www.springnutrition.org/sites/default/files/publications/briefs/spring_understandingpathways_brief_1.pdf . Accessed 12 Jan 2021.

[19] Kadiyala S, Harris J, Headey D, Yosef S, Gillespie S (2014). Agriculture and nutrition in India: mapping evidence to pathways. Ann N Y Acad Sci.

[20] Santeramo FG (2021). Exploring the link among food loss, waste and food security: what the research should focus on?. Agric Food Secur.

[21] Dizon F, Josephson A, Raju D (2021). Pathways to better nutrition in South Asia: evidence on the effects of food and agricultural interventions. Glob Food Sec..

[22] von Braun J, Afsana K, Fresco LO, Hassan M (2021). Food systems: seven priorities to end hunger and protect the planet. Nature.

[23] Masset E, Haddad L, Cornelius A, Isaza-Castro J (2012). Effectiveness of agricultural interventions that aim to improve nutritional status of children: systematic review. BMJ.

[24] Ruel MT, Quisumbing AR, Balagamwala M (2018). Nutrition-sensitive agriculture: what have we learned so far?. Glob Food Sec.

[25] Sharma IK, Di Prima S, Essink D, Broerse JEW (2020). Nutrition-sensitive agriculture: a systematic review of impact pathways to nutrition outcomes. Adv Nutr.

[26] Di Prima S, Wright EP, Sharma IK, Syurina E, Broerse JEW (2021). Implementation and scale-up of nutrition-sensitive agriculture in low- and middle-income countries: a systematic review of what works, what doesn’t work and why. Glob Food Sec..

[27] Nordhagen S, Nielsen J, van Mourik T, Smith E, Klemm R (2019). Fostering CHANGE: Lessons from implementing a multi-country, multi-sector nutrition-sensitive agriculture project. Eval Program Plann..

[28] Harris-Fry H, O’Hearn M, Pradhan R, Krishnan S, Nair N, Rath S (2020). How to design a complex behaviour change intervention: experiences from a nutrition-sensitive agriculture trial in rural India. BMJ Glob Heal..

[29] Green L, Kreuter M (2005). Health program planning: an educational and ecological approach.

[30] Lodenstein E, Dieleman M, Gerretsen B, Broerse JE (2013). A realist synthesis of the effect of social accountability interventions on health service providers’ and policymakers’ responsiveness. Syst Rev.

[31] Rawat R, Nguyen PH, Ali D, Saha K, Alayon S, Kim SS (2013). Learning how programs achieve their impact: embedding theory-driven process evaluation and other program learning mechanisms in alive & thrive. Food Nutr Bull.

[32] Olney DK, Vicheka S, Kro M, Chakriya C, Kroeun H, Hoing LS (2013). Using program impact pathways to understand and improve program delivery, utilization, and potential for impact of Helen Keller International’s Homestead Food Production Program in Cambodia. Food Nutr Bull.

[33] Bazeley P (2013). Qualitative data analysis: practical strategies.

[34] Baliki G, Brück T, Schreinemachers P, Uddin MN (2019). Long-term behavioural impact of an integrated home garden intervention: evidence from Bangladesh. Food Secur.

[35] Bernet T, Kurbanalieva S, Pittore K, Zilly B, Luttikholt L, Eyhorn F (2018). Nutrition-sensitive agriculture interventions in mountain areas—lessons learned from a 5-country project to upscale best practices. Mt Res Dev.

[36] Muehlhoff E, Wijesinha-Bettoni R, Westaway E, Jeremias T, Nordin S, Garz J (2017). Linking agriculture and nutrition education to improve infant and young child feeding: lessons for future programmes. Matern Child Nutr.

[37] Omer A, Mulualem D, Classen H, Vatanparast H, Whiting SJ (2018). A community poultry intervention to promote egg and eggshell powder consumption by young children in Halaba Special Woreda, SNNPR, Ethiopia. J Agric Sci.

[38] Sako B, Leerlooijer JN, Lelisa A, Hailemariam A, Brouwer ID, Tucker Brown A (2018). Exploring barriers and enablers for scaling up a community-based grain bank intervention for improved infant and young child feeding in Ethiopia: a qualitative process evaluation. Matern Child Nutr.

[39] Pradhan A, Sathanandhan R, Panda AK, Wagh R (2018). Improving household diet diversity through promotion of nutrition gardens in India. Am J Food Sci Nutr.

[40] Schreinemachers P, Patalagsa MA, Uddin N (2016). Impact and cost-effectiveness of women’s training in home gardening and nutrition in Bangladesh. J Dev Eff.

[41] Marquis GS, Colecraft EK, Kanlisi R, Aidam BA, Atuobi-Yeboah A, Pinto C (2018). An agriculture–nutrition intervention improved children’s diet and growth in a randomized trial in Ghana. Matern Child Nutr.

[42] Hagenimana V, Low J, Anyango M, Kurz K, Gichuki ST, Kabira J (2001). Enhancing vitamin A intake in young children in Western Kenya: Orange-fleshed sweet potatoes and women farmers can serve as key entry points. Food Nutr Bull.

[43] Roche ML, Sako B, Osendarp SJM, Adish AA, Tolossa AL (2017). Community-based grain banks using local foods for improved infant and young child feeding in Ethiopia. Matern Child Nutr.

[44] Miller LC, Neupane S, Joshi N, Lohani M, Rogers BL, Neupane S (2019). Multisectoral community development in Nepal has greater effects on child growth and diet than nutrition education alone. Public Health Nutr.

[45] Olney DK, Pedehombga A, Ruel MT, Dillon A (2015). A 2-year integrated agriculture and nutrition and health behavior change communication program targeted to women in Burkina Faso reduces anemia, wasting, and diarrhea in children 3–12.9 months of age at baseline: a cluster-randomized controlled trial. J Nutr.

[46] Worsley A (2002). Nutrition knowledge and food consumption: can nutrition knowledge change food behaviour?. Asia Pac J Clin Nutr.

[47] Low JW, Arimond M, Osman N, Cunguara B, Zano F, Tschirley D (2007). Ensuring the supply of and creating demand for a biofortified crop with a visible trait: lessons learned from the introduction of orange-fleshed sweet potato in drought-prone areas of Mozambique. Food Nutr Bull.

[48] Okello JJ, Kwikiriza N, Muoki P, Wambaya J, Heck S (2019). Effect of intensive agriculture-nutrition education and extension program adoption and diffusion of biofortified crops. J Agric Food Inf.

[49] Bauchspies WK, Diarra F, Rattunde F, Weltzien E (2017). “An Be Jigi”: collective cooking, whole grains, and technology transfer in Mali. Facets.

[50] De Brauw A, Eozenou P, Gilligan DO, Hotz C, Kumar N, Meenakshi JV (2018). Biofortification, crop adoption and health information: Impact pathways in Mozambique and Uganda. Am J Agric Econ.

[51] Kalavathi S, Krishnakumar VP, Thomas RJ, Thomas GV, George ML (2010). Improving food and nutritional security of small and marginal coconut growers through diversification of crops and enterprises. J Agric Rural Dev Trop Subtrop.

[52] Nordhagen S, Thiam K, Sow S (2019). The sustainability of a nutrition-sensitive agriculture intervention: a case study from urban Senegal. Food Secur.

[53] Nielsen JN, Olney DK, Ouedraogo M, Pedehombga A, Rouamba H, Yago-Wienne F (2018). Process evaluation improves delivery of a nutrition-sensitive agriculture programme in Burkina Faso. Matern Child Nutr.

[54] Cole DC, Levin C, Loechl C, Thiele G, Grant F, Girard AW (2016). Planning an integrated agriculture and health program and designing its evaluation: experience from Western Kenya. Eval Program Plann.

[55] Talukder A, Kiess L, Huq N, De Pee S, Darnton-Hill I, Bloem MW (2000). Increasing the production and consumption of vitamin A-rich fruits and vegetables: lessons learned in taking the Bangladesh homestead gardening programme to a national scale. Food Nutr Bull.

[56] Nordhagen S, Klemm R (2018). Implementing small-scale poultry-for-nutrition projects: successes and lessons learned. Matern Child Nutr.

[57] Kerr RB, Chilanga E, Nyantakyi-Frimpong H, Luginaah I, Lupafya E (2016). Integrated agriculture programs to address malnutrition in northern Malawi. BMC Public Health.

[58] Boedecker J, Odhiambo Odour F, Lachat C, Van Damme P, Kennedy G, Termote C (2019). Participatory farm diversification and nutrition education increase dietary diversity in Western Kenya. Matern Child Nutr.

[59] Haselow NJ, Stormer A, Pries A (2016). Evidence-based evolution of an integrated nutrition-focused agriculture approach to address the underlying determinants of stunting. Matern Child Nutr.

[60] Michaux KD, Hou K, Karakochuk CD, Whitfield KC, Ly S, Verbowski V (2019). Effect of enhanced homestead food production on anaemia among Cambodian women and children: a cluster randomized controlled trial. Matern Child Nutr.

[61] Girard AW, Grant F, Watkinson M, Okuku HS, Wanjala R, Cole D (2017). Promotion of orange-fleshed sweet potato increased Vitamin A intakes and reduced the odds of low retinol-binding protein among postpartum Kenyan women. J Nutr.

[62] Schreinemachers P, Bhattarai DR, Subedi GD, Acharya TP, Chen H, Yang R (2017). Impact of school gardens in Nepal: a cluster randomised controlled trial. J Dev Eff..

[63] Bushamuka VN, de Pee S, Talukder A, Kiess L, Panagides D, Taher A (2005). Impact of a homestead gardening program on household food security and empowerment of women in Bangladesh. Food Nutr Bull.

[64] Matturi K, Pain C (2016). Managing an integrated project—experiences from the realigning agriculture to improve nutrition project. Proj Manag Res Pract.

[65] Kjeldsberg C, Shrestha N, Patel M, Davis D, Mundy G, Cunningham K (2018). Nutrition-sensitive agricultural interventions and gender dynamics: a qualitative study in Nepal. Matern Child Nutr.

[66] Doocy S, Emerson J, Colantouni E, Strong J, Mansen KA, Caulfield LE (2018). Improving household food security in eastern Democratic Republic of the Congo: a comparative analysis of four interventions. Food Secur.

[67] Ayele Z, Peacock C (2003). Improving access to and consumption of animal source foods in rural households: the experiences of a women-focused goat development program in the highlands of Ethiopia. J Nutr.

[68] Gillespie S, Poole N, van den Bold M, Bhavani RV, Dangour AD, Shetty P (2019). Leveraging agriculture for nutrition in South Asia: what do we know, and what have we learned?. Food Policy.

[69] Ali M, Villa KM, Joshi J (2018). Health and hunger: nutrient response to income depending on caloric availability in Nepal. Agric Econ.

[70] Le Port A, Bernard T, Hidrobo M, Birba O, Rawat R, Ruel MT (2017). Delivery of iron-fortified yoghurt, through a dairy value chain program, increases hemoglobin concentration among children 24 to 59 months old in Northern Senegal: a cluster-randomized control trial. PLoS ONE.

